# The Monocyte-to-Lymphocyte Ratio Exhibits A Superior Prognostic Value in Patients with Newly Diagnosed Acute Coronary Syndrome

**DOI:** 10.31083/RCM39917

**Published:** 2025-10-30

**Authors:** Mi Lao, Huiling Liu, Xiaoping Cai, Yue Zhang, Tong Liu, Guangping Li, Kangyin Chen, Meng Yuan

**Affiliations:** ^1^Tianjin Key Laboratory of Ionic-Molecular Function of Cardiovascular Disease, Department of Cardiology, Tianjin Institute of Cardiology, The Second Hospital of Tianjin Medical University, 300211 Tianjin, China; ^2^Department of Cardiology, Binzhou People’s Hospital, 256600 Binzhou, Shandong, China; ^3^Department of Radiation Oncology, Binzhou People’s Hospital, 256600 Binzhou, Shandong, China

**Keywords:** acute coronary syndrome, major adverse cardiovascular events, novel inflammatory markers, prognosis, monocyte-lymphocyte ratio

## Abstract

**Background:**

Chronic inflammation critically influences atherosclerotic progression and plaque destabilization. This investigation assessed and compared six lymphocyte-derived inflammatory indices (neutrophil-to-lymphocyte ratio (NLR), monocyte–lymphocyte ratio (MLR), platelet–lymphocyte ratio (PLR), systemic immune–inflammation index (SII), systemic inflammatory response index (SIRI), systemic immune–inflammation response index (SIIRI)) for predicting major adverse cardiovascular events (MACEs) in treatment-naïve acute coronary syndrome (ACS) patients undergoing coronary angiography.

**Methods:**

This study enrolled 1120 patients with newly diagnosed ACS, in which the occurrence of MACEs was monitored. The predictive capacities of the included lymphocyte-derived inflammatory indices were evaluated through receiver operator characteristic (ROC) curve analysis with optimal cutoffs, supplemented by Cox proportional hazards modeling.

**Results:**

A total of 265 MACEs (23.66%) were recorded during the 64.20 ± 23.05-month follow-up. Multivariate Cox analyses identified an elevated MLR (hazard ratio (HR) = 2.880, 95% confidence interval (CI) 1.280–6.470; *p* < 0.001) that was independently associated with the occurrence of MACEs in patients with newly diagnosed ACS. The ROC comparisons revealed a superior discriminative capacity of the MLR versus clinical factors, with an optimal MLR cutoff at 0.304 (sensitivity 61.1%; specificity 78.8%). Patients with a high MLR (≥0.304) exhibited a 3.5-fold increased risk of MACEs compared to those with a low MLR (46.96% vs. 13.29%; risk ratio = 1.635, 95% CI 1.475–1.812; *p* < 0.001); these data were corroborated by divergent Kaplan–Meier curves (log-rank *p* < 0.001). Meanwhile, subgroup analyses confirmed the prognostic consistency of the MLR across high-risk populations (age >60 years, diabetes, hypertension), with elevated MLR subgroups demonstrating uniformly higher rates of MACEs (all *p* < 0.001).

**Conclusions:**

MLR outperformed conventional parameters and five novel lymphocyte-based inflammatory indices in predicting MACEs in ACS patients; thus, the MLR can be established as a robust predictive biomarker. The clinical utility of the MLR extends to risk stratification across key patient subgroups, suggesting potential integration into routine cardiovascular risk assessment protocols.

## 1. Introduction

Coronary heart disease (CHD) is the leading cause of mortality and morbidity worldwide [1]. Acute coronary syndrome (ACS) is one of the most severe manifestations of CHD [2]. ACS is characterized by acute myocardial ischemia because of the formation of intracoronary thrombi due to the rupture or erosion of unstable atherosclerotic plaques, and encompasses unstable angina (UA), ST-segment elevation myocardial infarction (STEMI), and non-ST-segment elevation myocardial infarction (NSTEMI) [3, 4]. Despite advances in revascularization techniques such as percutaneous coronary intervention (PCI), the prognosis of patients with ACS remains poor and unsatisfactory [5, 6]. Furthermore, the incidence rates of ACS in China have consistently increased over the past few decades [7]. Therefore, identifying risk factors associated with an adverse prognosis in patients with ACS is of paramount importance to identify patients at higher risk and to delineate personalized therapeutic strategies.

Inflammation is an important feature in all stages of atherosclerosis, including acute thrombotic complications and clinical events [8]. Inflammation is pivotal in the development and instability of the coronary plaques and contributes significantly to plaque rupture. Coronary plaques contain activated macrophages, which promote plaque rupture, arterial wall thrombosis, and vessel constriction [9]. Interleukin (IL)-17 is a pro-inflammatory cytokine that exerts a significant influence on atherosclerosis and ACS [10]. More than half of the patients with atherosclerotic cardiovascular disease are associated with systemic inflammation. The incidences of major adverse cardiovascular events (MACEs), heart failure (HF), and mortality increase significantly when the C-reactive protein (CRP) levels are ≥2 mg/L [11]. IL-6 is a pro-inflammatory cytokine primarily secreted by macrophages and T cells. Plasma IL-6 levels show significant prognostic value in patients with ACS [12]. Moreover, plasma IL-6 levels are positively correlated with CRP levels in patients with ACS [13]. In addition to inflammatory factors such as RCP, higher leukocyte counts (e.g., neutrophils, monocytes) in newly diagnosed ACS patients have been shown to significantly correlate with increased mortality [14]. However, the most optimal inflammatory predictor indicator that is both simple and practical remains uncertain. There is an urgent need to characterize novel clinical biomarkers for the routine and accurate estimation of the chronic inflammatory status of patients. Recent studies have shown that, compared to simple blood cell counts, ratios based on blood cells (such as the monocyte-to-lymphocyte ratio (MLR)) demonstrate significantly more reliable performance in predicting MACEs [15]. In a cohort of ACS patients undergoing PCI, multivariate Cox regression analysis demonstrated that MACEs were significantly and independently associated with five hematological inflammatory indices, namely, neutrophil-to-lymphocyte ratio (NLR), platelet-to-lymphocyte ratio (PLR), MLR, systemic immune-inflammation index (SII), and systemic inflammation response index (SIRI) [16]. In a recent meta-analysis, higher SII levels demonstrated significant independent prognostic value for MACEs and all-cause mortality in patients with ACS [17]. As a novel inflammatory index derived from lymphocyte parameters, the systemic immune-inflammatory response index (SIIRI) has been found to exhibit independent prognostic value for MACEs in newly diagnosed coronary artery disease (CAD) patients [18]. The SIIRI is also an independent predictor of severe CAD [19]. The SIIRI emerged as an independent prognostic predictor of MACEs in newly diagnosed CAD patients after comparative analysis of established inflammatory indices (NLR, PLR, MLR, SII, and SIRI) using multivariable adjusted models [20]. However, compared to MLR and SIIRI, only SIIRI was a predictor of severe CAD [21]. Currently, the clinical values of novel inflammatory biomarkers such as NLR, PLR, MLR, SII, SIRI, and SIIRI are inconclusive for predicting adverse clinical outcomes in newly diagnosed ACS patients. Furthermore, the definitions of MACEs vary widely across observational studies, with only 8.6% of the studies matching the traditional three-point MACE definitions and none of the studies matching the four-point or five-point MACE definitions [22].

In this study, we compared the prognostic values of six different inflammatory markers to identify indicators that can precisely predict MACEs in patients with newly diagnosed ACS. The aim was to identify the best inflammatory indicator that can be used for clinical monitoring and strategizing personalized treatment plans for patients with ACS to improve their prognosis and quality of life.

## 2. Materials and Methods

### 2.1 Study Population

**Fig. 1.**
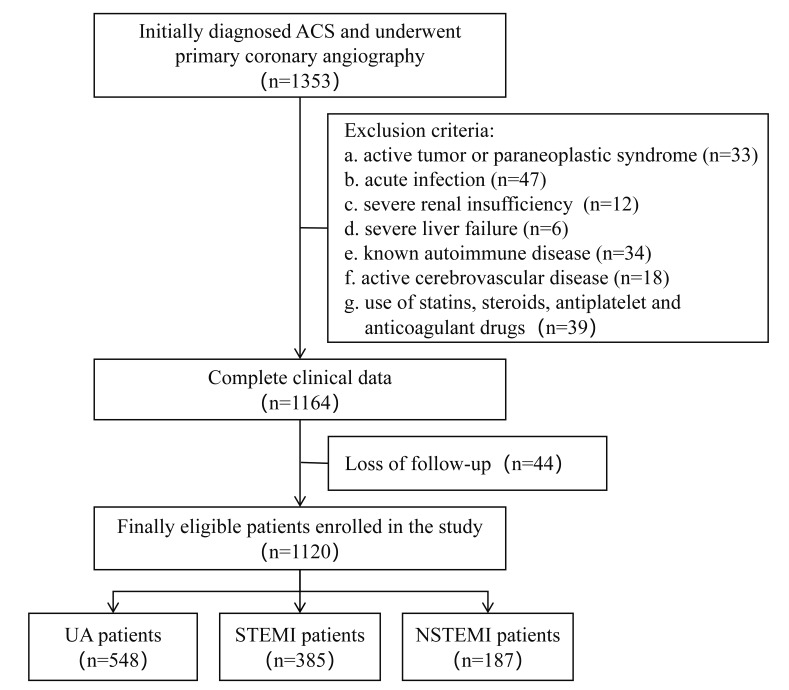
**Flowchart of the study cohort**. Abbreviations: ACS, acute coronary syndrome; UA, unstable angina; STEMI, ST-segment elevation myocardial infarction; NSTEMI, non-ST-segment elevation myocardial infarction.

We retrospectively enrolled 1120 newly diagnosed ACS patients who underwent primary coronary angiography and were diagnosed with ACS at our hospital from August 2018 to December 2020. All ACS patients underwent diagnostic coronary angiography, and PCI was determined based on the degree of coronary artery stenosis. The inclusion criteria were as follows: (1) ACS diagnosis according to the published 2023 ESC guidelines [23]; (2) 18 years old or older; and (3) availability of complete clinical data from the electronic medical records. The exclusion criteria were as follows: (1) active tumor or paraneoplastic syndrome; (2) acute infection; (3) severe renal insufficiency (estimated glomerular filtration rate <30 mL/min/1.73 m^2^); (4) severe liver failure; (5) known autoimmune disease; (6) active cerebrovascular disease; and (7) use of statins, steroids, antiplatelet and anticoagulant drugs. The flow chart of the patient selection strategy is shown in Fig. 1. This retrospective study was conducted in accordance with the Declaration of Helsinki guidelines. Ethical approval was obtained from the Ethics Committee of Binzhou People’s Hospital. The requirement for written informed consent was waived because of the retrospective nature of this study.

### 2.2 Clinical and Laboratory Data

We collected baseline clinical data, laboratory test results, and coronary angiography findings from the electronic medical records. The basic clinical information of the patients included age, gender, smoking history, diabetes mellitus, hypertension, dyslipidemia, atrial fibrillation (AF), and family history of coronary heart disease. Laboratory examinations before diagnostic coronary angiography included complete blood cell counts, blood glucose, total cholesterol, low-density lipoprotein (LDL), high-density lipoprotein (HDL), triglyceride, serum creatinine, glomerular filtration rate, and other biochemical tests. The basic coronary angiography data included lesion status of the left main coronary artery, right coronary artery, left anterior descending artery, and left circumflex artery. To assess the systemic inflammatory biomarkers, six hematological indices were calculated based on differential complete blood cell counts. The NLR was calculated by dividing the absolute neutrophil counts by the absolute lymphocyte counts. The PLR was calculated by dividing the platelet counts by the absolute lymphocyte counts. The MLR was calculated by dividing the absolute monocyte counts by the absolute lymphocyte counts. The three composite indices were formulated as follows: SII = platelet counts × NLR; SIRI = monocyte counts × NLR; and SIIRI = platelet counts × monocyte counts × NLR.

### 2.3 Follow-Up

Patients were scheduled for follow-up assessments every six months after hospital discharge. The follow-up was mainly carried out through telephone, outpatient review, or inpatient observation, with a primary focus on documenting MACEs, including non-fatal myocardial infarction, non-fatal stroke, all-cause mortality, UA, and HF. The observation period continued until the occurrence of the first MACEs or the predetermined study termination date of January 31, 2025, whichever occurred first.

### 2.4 Statistical Analysis

Continuous variables were presented as mean ± standard deviation or median (25th to 75th percentile). The *t*-test or the Mann-Whitney U test was used to determine the statistically significant differences in the continuous variables between two groups. Categorical variables are displayed as frequencies and percentages. The χ^2^ or Fisher’s exact tests were used to determine the significance of categorical variables between the two groups. The optimum cut-off values for the predictive characteristics were based on the Youden index, which was derived from the receiver operating characteristic (ROC) curves. Least absolute shrinkage and selection operator (LASSO) regression with 10-fold cross-validation was used to select the most relevant variables. Univariable Cox proportional hazards regression was used to identify factors potentially associated with MACEs. Variables with *p*-value < 0.1 in the univariable analysis were entered into a multivariable Cox proportional hazards regression model. Kaplan-Meier survival curves were used to analyze the prognostic differences between groups. Two-tailed *p*-values < 0.05 were considered statistically significant. All statistical analyses were performed using SPSS version 25.0 (IBM, Armonk, NY, USA), R version 4.2.2 (R Foundation for Statistical Computing, Vienna, Austria), and GraphPad Prism version 8.0 (GraphPad Software, La Jolla, CA, USA). Missing cases among selected variables were excluded in this study.

## 3. Results

### 3.1 Patient Characteristics

This study included 1120 newly diagnosed ACS patients with a mean age of 61.67 ± 10.63 years. Among these, 63.90% were males, 18.75% had diabetes, 56.52% had hypertension, and 32.41% had a smoking history. During a median follow-up period of 64.20 ± 23.05 months, 265 (23.66%) patients experienced MACEs. Among these patients, 183 (11.34%) were diagnosed with UA, 35 (3.13%) with acute myocardial infarction, 28 (2.50%) with HF, 16 (1.43%) with stroke, and 3 (0.27%) with a cardiovascular death. Table 1 outlines the baseline characteristics of the study cohort. Compared with the event-free patients, patients with MACEs demonstrated a higher prevalence of male gender (70.20% vs. 61.90%, *p* = 0.015), diabetes mellitus (26.42% vs. 16.37%, *p* < 0.001), and AF (3.02% vs. 1.17%, *p* = 0.036). However, we did not observe any statistically significant differences between the two groups regarding the proportion of patients with a history of smoking or alcohol consumption.

**Table 1.** **Baseline characteristics of 1120 patients with newly diagnosed ACS**.

		ALL (N = 1120)	No such event (N = 855)	MACEs (N = 265)	χ^2^/Z	*p*-value
Diagnosis					78.462	<0.001
	UA, n (%)	548 (48.92)	480 (56.14)	68 (25.66)		
	STEMI, n (%)	385 (34.38)	261 (30.53)	124 (46.79)		
	NSTEMI, n (%)	187 (16.70)	114 (13.33)	73 (27.55)		
Age (years)		63 (54, 70)	63 (54, 69)	65 (56, 70)	–3.475	0.001
Male sex, n (%)		716 (63.93)	530 (61.90)	186 (70.20)	5.899	0.015
Diabetes mellitus, n (%)		210 (18.75)	140 (16.37)	70 (26.42)	13.388	<0.001
Hypertension, n (%)		633 (56.52)	463 (54.15)	170 (64.15)	8.230	0.004
New diagnosis dyslipidemia, n (%)		42 (3.75)	34 (3.98)	8 (3.02)	0.514	0.473
Current smoker, n (%)		363 (32.41)	281 (32.87)	82 (30.94)	0.341	0.559
Current drinkers, n (%)		128 (11.43)	105 (12.28)	23 (8.68)	2.592	0.107
Stroke, n (%)		92 (8.21)	66 (7.72)	26 (9.81)	1.174	0.279
AF, n (%)		18 (1.61)	10 (1.17)	8 (3.02)	4.375	0.036
Family history of CAD, n (%)		24 (2.14)	18 (2.11)	6 (2.26)	0.024	0.876
Syncope, n (%)		11 (0.98)	5 (0.58)	6 (2.26)	5.867	0.015
Tumor, n (%)		20 (1.79)	16 (1.87)	4 (1.51)	0.151	0.698
Heart rate		74 (66, 82)	72 (65, 81)	76 (68, 88)	–3.202	0.001
Systolic pressure		140 (123, 156)	141 (125, 157)	136 (121, 156)	–1.957	0.050
Diastolic pressure		85 (75, 95)	85 (75, 94)	85 (74, 96)	–0.111	0.911
Coronary lesion type					33.800	<0.001
	Left main coronary artery disease, n (%)	57 (5.09)	36 (4.21)	21 (7.92)		
	Polyvascular disease, n (%)	627 (55.98)	447 (52.28)	180 (67.92)		
	Branch lesions, n (%)	436 (38.93)	372 (43.51)	64 (24.15)		
NLR		2.64 (1.75, 4.20)	2.42 (1.60, 3.50)	4.21 (2.50, 8.10)	–11.043	<0.001
PLR		129.38 (97.85, 169.72)	124.26 (95.50, 160.30)	147.37 (114.50, 205.90)	–6.998	<0.001
MLR		0.24 (0.18, 0.33)	0.22 (0.20, 0.30)	0.35 (0.23, 0.52)	–11.943	<0.001
SII		574.24 (380.78, 976.65)	515.67 (355.40, 784.50)	1077.58 (548.50, 1857.60)	–12.128	<0.001
SIRI		1.08 (0.64, 2.05)	0.92 (0.60, 1.50)	2.50 (1.16, 4.73)	–13.582	<0.001
SIIRI		235.95 (135.71, 462.70)	195.53 (122.10, 342.50)	628.46 (259.34, 1110.98)	–14.128	<0.001
Fasting blood glucose (mmol/L)		5.73 (5.04, 7.02)	5.59 (5.02, 6.69)	6.05 (5.15, 7.81)	–4.343	<0.001
Urea nitrogen (mmol/L)		4.87 (4.00, 5.90)	4.75 (3.90, 5.71)	5.20 (4.34, 6.40)	–4.632	<0.001
Creatinine (umol/L)		68 (58, 79)	67 (58, 78)	72 (59, 82)	–2.597	0.009
Uric acid (umol/L)		303 (246, 366)	303 (247, 366)	303 (246, 368)	–0.066	0.948
Albumin (g/L)		43.10 (40.30, 45.92)	43.50 (40.70, 46.01)	42.07 (39.17, 45.40)	–3.781	<0.001
Total cholesterol (mmol/L)		4.49 (3.81, 5.24)	4.47 (3.79, 5.19)	4.64 (3.88, 5.41)	–1.794	0.073
Triglycerides (mmol/L)		1.44 (1.02, 2.02)	1.47 (1.03, 2.04)	1.38 (1.01, 1.92)	–1.306	0.192
High-density lipoprotein (mmol/L)		1.10 (0.93, 1.30)	1.11 (0.94, 1.30)	1.09 (0.92, 1.28)	–1.082	0.279
Low-density lipoprotein (mmol/L)		2.66 (2.07, 3.35)	2.62 (2.03, 3.33)	2.79 (2.19, 3.51)	–2.583	0.010
Lipoprotein (mg/dL)		16.80 (8.25, 33.70)	16.30 (8.00, 32.60)	18.40 (9.00, 38.80)	–1.412	0.158

Abbreviations: UA, unstable angina; STEMI, ST-segment elevation myocardial infarction; NSTEMI, non-ST-segment elevation myocardial infarction; AF, Atrial fibrillation; NLR, neutrophil-to-lymphocyte ratio; PLR, platelet-to-lymphocyte ratio; MLR, monocyte-to-lymphocyte ratio; SII, systemic immune-inflammation index; SIRI, systemic inflammation response index; SIIRI, systemic immune-inflammatory response index; MACEs, major adverse cardiovascular events.

### 3.2 LASSO and Cox Regression Analysis

We performed LASSO regression with 10-fold cross-validation on 33 candidate variables. The optimal regularization parameter (λ) was selected under the minimum mean squared error criterion (λ = 0.023), yielding 13 predictors with non-zero coefficients. To determine the independent predictors of MACEs in patients newly diagnosed with ACS, univariate and multivariate Cox regression analyses were conducted on the retained variables (Table 2). Cox regression analysis demonstrated that age, hypertension, diabetes mellitus, diagnostic status, coronary lesion type, LDL, and HDL levels were independent predictors of MACEs. Furthermore, inflammatory biomarker MLR (hazard ratio (HR) 2.880, 95% confidence interval (CI) 1.280–6.470, *p* < 0.001) was independently associated with the occurrence of MACEs.

**Table 2.** **The univariable and multivariable Cox regression analysis**.

	Univariable Cox regression		Multivariable Cox regression	
	HR (95% CI)	*p*-value	HR (95% CI)	*p*-value
Diagnosis	1.870 (1.609–2.173)	<0.001	1.710 (1.445–2.024)	<0.001
Age	1.021 (1.009–1.033)	0.001	1.012 (1.000–1.025)	0.049
Diabetes mellitus	1.737 (1.322–2.284)	<0.001	1.576 (1.185–2.097)	0.002
Hypertension	1.456 (1.133–1.872)	0.003	1.552 (1.200–2.011)	0.001
Tumor	0.861 (0.321–2.312)	0.766		
Atrial fibrillation	2.004 (0.991–4.051)	0.053	0.717 (0.340–1.510)	0.382
Syncope	2.432 (1.082–5.465)	0.026	1.306 (0.574–2.970)	0.525
Heart rate	1.018 (1.010–1.026)	<0.001	1.000 (1.000–1.010)	0.252
Coronary lesion type	0.544 (0.442–0.669)	<0.001	0.702 (0.555–0.887)	0.003
MLR	15.473 (10.859–22.048)	<0.001	2.880 (1.280–6.470)	<0.001
Creatinine	1.006 (1.003–1.008)	<0.001	1.000 (1.000–1.010)	0.415
High-density lipoprotein	1.064 (1.025–1.105)	<0.001	1.060 (1.020–1.090)	0.001
Low-density lipoprotein	1.140 (1.031–1.260)	0.011	1.130 (1.020–1.240)	0.019

Abbreviations: HR, hazard ratio; CI, confidence interval; NLR, neutrophil-to-lymphocyte ratio; MLR, monocyte-to-lymphocyte ratio.

### 3.3 ROC Curve Analysis and Optimal Cut-Off Values for the Indicators

**Fig. 2.**
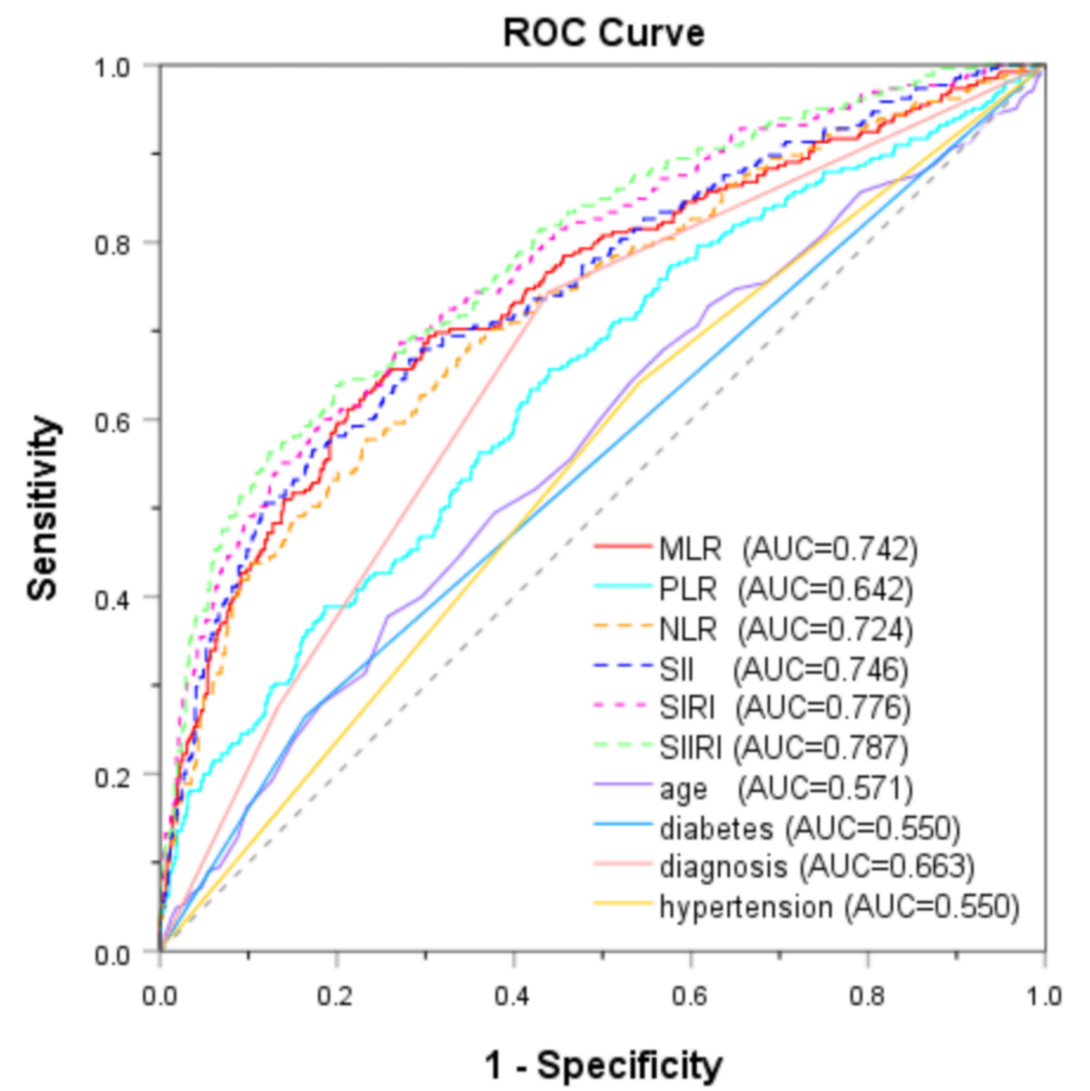
**ROC curves of inflammatory biomarkers and clinical factors for predicting MACEs**. ROC, receiver operator characteristic; MACEs, major adverse cardiovascular events; AUC, area under the curve; MLR, monocyte-to-lymphocyte ratio; PLR, platelet-to-lymphocyte ratio; NLR, neutrophil-to-lymphocyte ratio; SII, systemic immune-inflammation index; SIRI, systemic inflammation response index; SIIRI, systemic immune-inflammatory response index.

During a mean follow-up period of 64.20 ± 23.05 months, MACEs occurred in 265 (23.66%) patients. The ROC curve for predicting MACEs in ACS patients using six inflammatory biomarkers and selected clinical factors is shown in Fig. 2. MLR demonstrated superior diagnostic performance compared to the PLR (z = 5.626, *p* < 0.001), age (z = 7.016, *p* < 0.001), diabetes (z = 7.813, *p* < 0.001), hypertension (z = 7.455, *p* < 0.001), or diagnosis (z = 3.649, *p* < 0.001), as determined by the DeLong test. Furthermore, the diagnostic performance of MLR did not show a significant statistical difference from that of NLR (z = 1.351, *p* = 0.177) or SII (z = 0.237, *p* = 0.813), but was inferior to SIRI (z = 3.303, *p* = 0.001) or SIIRI (z = 3.643, *p* < 0.001). Based on the ROC curve analysis, the optimal MLR cut-off value was 0.304 for predicting MACEs. The patients were categorized into the high-MLR and low-MLR groups based on the optimal MLR cut-off value. Then, the correlations of clinical factors and inflammatory markers between the high-MLR and the low-MLR groups were evaluated as shown in Table 3. Table 4 details the optimal cut-off values, 95% CI, sensitivity, specificity, positive predictive value, and negative predictive value for each biomarker. **Supplementary Fig. 1** and **Supplementary Table 1** present the diagnostic performance of inflammatory factors and selected clinical factors in predicting HF.

**Table 3.** **Baseline characteristics between the high-MLR and the low-MLR groups**.

		ALL (N = 1120)	MLR <0.304 (N = 775)	MLR ≥0.304 (N = 345)	χ^2^/Z	*p*-value
Diagnosis					148.107	<0.001
	UA, n (%)	548 (48.92)	470 (60.65)	78 (22.61)		
	STEMI, n (%)	385 (34.38)	189 (24.39)	196 (56.81)		
	NSTEMI, n (%)	187 (16.70)	116 (14.97)	71 (20.58)		
Age (years)		63 (54, 70)	62 (54, 68)	65 (55, 71)	–3.495	<0.001
Male sex, n (%)		716 (63.93)	455 (58.71)	261 (75.65)	29.717	<0.001
Diabetes mellitus, n (%)		210 (18.75)	152 (19.61)	58 (16.81)	0.317	0.574
Hypertension, n (%)		633 (56.52)	442 (57.03)	191 (55.36)	0.271	0.603
New diagnosis dyslipidemia, n (%)		42 (3.75)	35 (4.52)	7 (2.03)	4.091	0.043
Current smoker, n (%)		363 (32.41)	251 (32.39)	112 (32.46)	0.001	0.980
Current drinkers, n (%)		128 (11.43)	88 (11.35)	40 (11.59)	0.014	0.907
Stroke, n (%)		92 (8.21)	58 (7.48)	34 (9.86)	1.780	0.182
AF, n (%)		18 (1.61)	7 (0.90)	11 (3.19)	7.884	0.005
Family history of CAD, n (%)		24 (2.14)	17 (2.19)	7 (2.03)	0.031	0.861
Syncope		11 (0.98)	5 (0.65)	6 (1.74)	2.938	0.087
Tumor		20 (1.79)	18 (2.32)	2 (0.58)	4.135	0.042
Heart rate		74 (66, 82)	72 (65, 80)	76 (67, 86)	–7.037	<0.001
Systolic pressure		140 (123, 156)	143 (128, 159)	130 (117, 150)	–7.014	<0.001
Diastolic pressure		85 (75, 95)	86 (76, 95)	83 (73, 93)	–2.646	0.008
Coronary artery disease					13.856	0.001
	Left main coronary artery disease, n (%)	57 (5.09)	40 (5.16)	17 (4.93)		
	Polyvascular disease, n (%)	627 (55.98)	406 (52.39)	221 (64.06)		
	Branch lesions, n (%)	436 (38.93)	329 (42.45)	101 (31.01)		
NLR		2.64 (1.75, 4.20)	2.14 (1.50, 2.80)	5.14 (3.40, 8.30)	–20.999	<0.001
PLR		129.38 (97.85, 169.72)	115.06 (92.50, 146.80)	163.25 (131.00, 228.30)	–13.546	<0.001
MLR		0.24 (0.18, 0.33)	0.20 (0.16, 0.24)	0.41 (0.34, 0.55)	–26.750	<0.001
SII		574.24 (380.78, 976.65)	459.72 (334.20, 666.00)	1089.26 (730.50, 1807.70)	–19.006	<0.001
SIRI		1.08 (0.64, 2.05)	0.77 (0.50, 1.20)	2.83 (1.90, 4.70)	–24.155	<0.001
SIIRI		235.95 (135.71, 462.70)	168.77 (114.20, 272.20)	663.56 (387.60, 1077.30)	–22.124	<0.001
Fasting blood glucose (mmol/L)		5.73 (5.04, 7.02)	5.61 (5.03, 6.82)	5.91 (5.05, 7.35)	–2.210	0.027
Urea_nitrogen (mmol/L)		4.87 (4.00, 5.90)	4.71 (3.90, 5.70)	5.10 (4.20, 6.37)	–3.660	<0.001
Creatinine (umol/L)		68 (58, 79)	67 (57, 77)	72 (62, 82)	–4.893	<0.001
Uric_acid (umol/L)		303 (246, 366)	302 (246, 365)	310 (248, 372)	–0.471	0.637
Albumin (g/L)		43.10 (40.30, 45.92)	43.61 (41.00, 46.30)	41.70 (38.90, 44.30)	–6.915	<0.001
Total cholesterol (mmol/L)		4.49 (3.81, 5.24)	4.52 (3.89, 5.28)	4.43 (3.73, 5.13)	–2.235	0.025
Triglycerides (mmol/L)		1.44 (1.02, 2.02)	1.51 (1.05, 2.10)	1.32 (0.97, 1.80)	–3.829	<0.001
High-density lipoprotein (mmol/L)		1.10 (0.93, 1.30)	1.11 (0.94, 1.30)	1.08 (0.90, 1.30)	–1.727	0.084
Low-density lipoprotein (mmol/L)		2.66 (2.07, 3.35)	2.69 (2.08, 3.38)	2.63 (2.02, 3.26)	–1.181	0.238
Lipoprotein (mg/dL)		16.80 (8.25, 33.70)	16.60 (7.80, 34.20)	17.60 (9.15, 32.95)	–1.022	0.307
MACEs		265 (23.66)	103 (13.29)	162 (46.96)	149.801	<0.001

Abbreviations: UA, unstable angina; STEMI, ST-segment elevation myocardial infarction; NSTEMI, non-ST-segment elevation myocardial infarction; AF, Atrial fibrillation; NLR, neutrophil-to-lymphocyte ratio; PLR, platelet-to-lymphocyte ratio; MLR, monocyte-to-lymphocyte ratio; SII, systemic immune-inflammation index; SIRI, systemic inflammation response index; SIIRI, systemic immune-inflammatory response index; MACEs, major adverse cardiovascular events; CAD, coronary artery disease.

**Table 4.** **Diagnostic performances of various biomarkers in predicting MACEs among ACS patients**.

Model	Cut-off value	*p*-value	AUC (95% CI)	SEN	SPE	PPV	NPV
Age	68 years	0.002	0.571 (0.530–0.611)	0.377	0.743	0.313	0.794
Age	65 years	0.009	0.558 (0.529–0.588)	0.494	0.622	0.289	0.799
Hypertension	Yes	0.007	0.550 (0.511–0.589)	0.642	0.458	0.269	0.805
Diagnosis	MI	<0.001	0.663 (0.635–0.691)	0.743	0.561	0.344	0.876
Diabetes mellitus	Yes	0.026	0.550 (0.509–0.591)	0.264	0.836	0.333	0.786
MLR	0.304	<0.001	0.742 (0.705–0.779)	0.611	0.788	0.470	0.867
HDL	1.15 mmol/L	0.283	0.522 (0.492–0.552)	0.646	0.423	0.259	0.796
LDL	2.73 mmol/L	0.009	0.552 (0.523–0.582)	0.543	0.552	0.273	0.796

Abbreviations: AUC, area under the curve; CI, confidence interval; SEN, Sensitivity; SPE, Specificity; PPV, positive predictive value; NPV, negative predictive value; MI, myocardial infarction; MLR, monocyte-to-lymphocyte ratio; PLR, platelet-to-lymphocyte ratio; HDL, High-density lipoprotein; LDL, Low-density lipoprotein.

### 3.4 Follow-Up and Survival Analysis

**Fig. 3.**
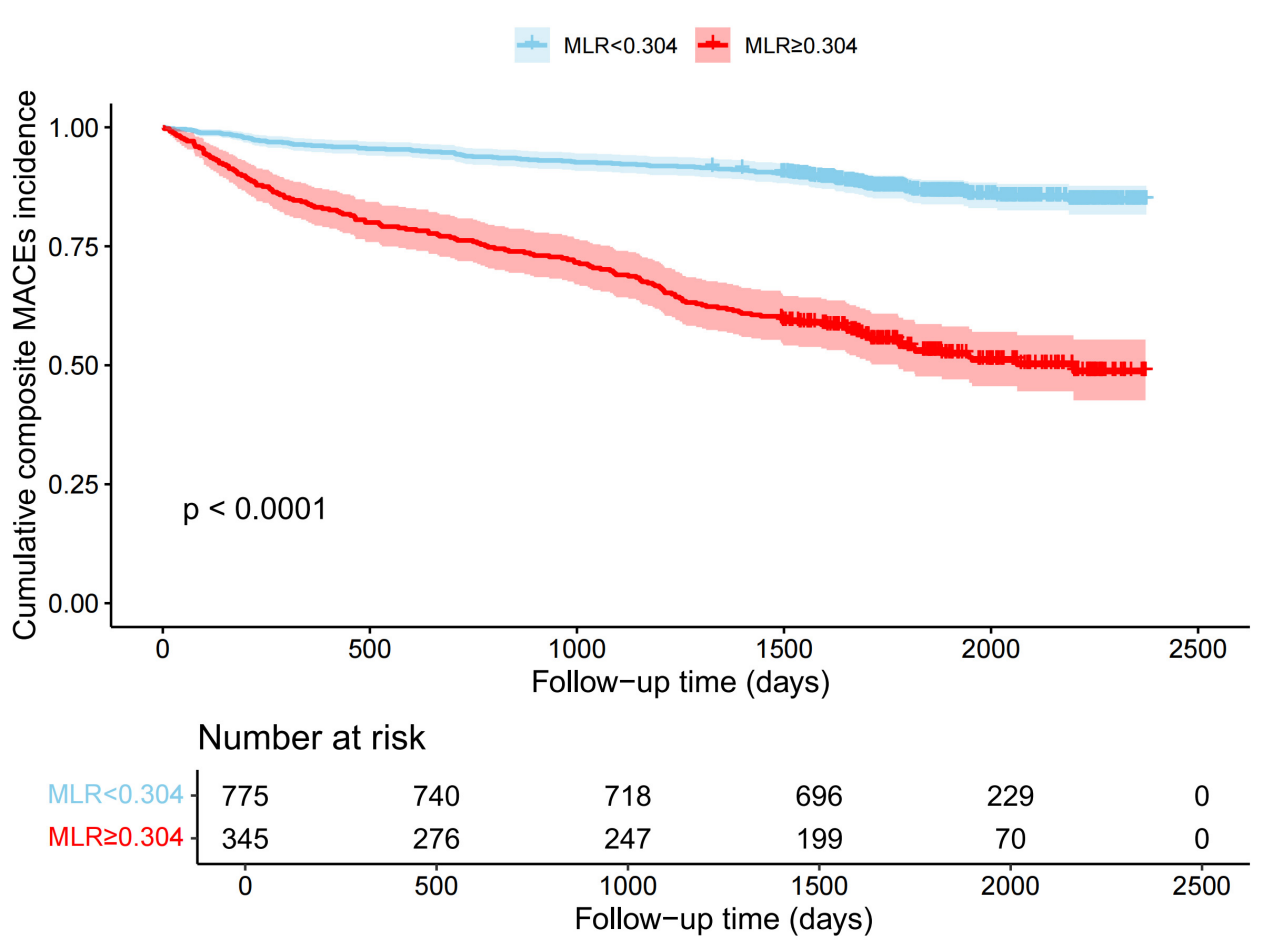
**Kaplan-Meier survival curve analysis of patients with ACS stratified according to MLR**. MLR, monocyte-lymphocyte ratio.

As shown in Table 3, patients in the high-MLR group demonstrated a significantly higher incidence of MACEs compared to the low-MLR group (46.96% vs. 13.29%; risk ratio 1.635, 95% CI 1.475–1.812, *p* < 0.001). Analysis of Kaplan-Meier survival curves demonstrated that the event-free survival probability was significantly higher in the low-MLR group compared to the high-MLR group (log-rank *p* < 0.001) (Fig. 3).

**Fig. 4.**
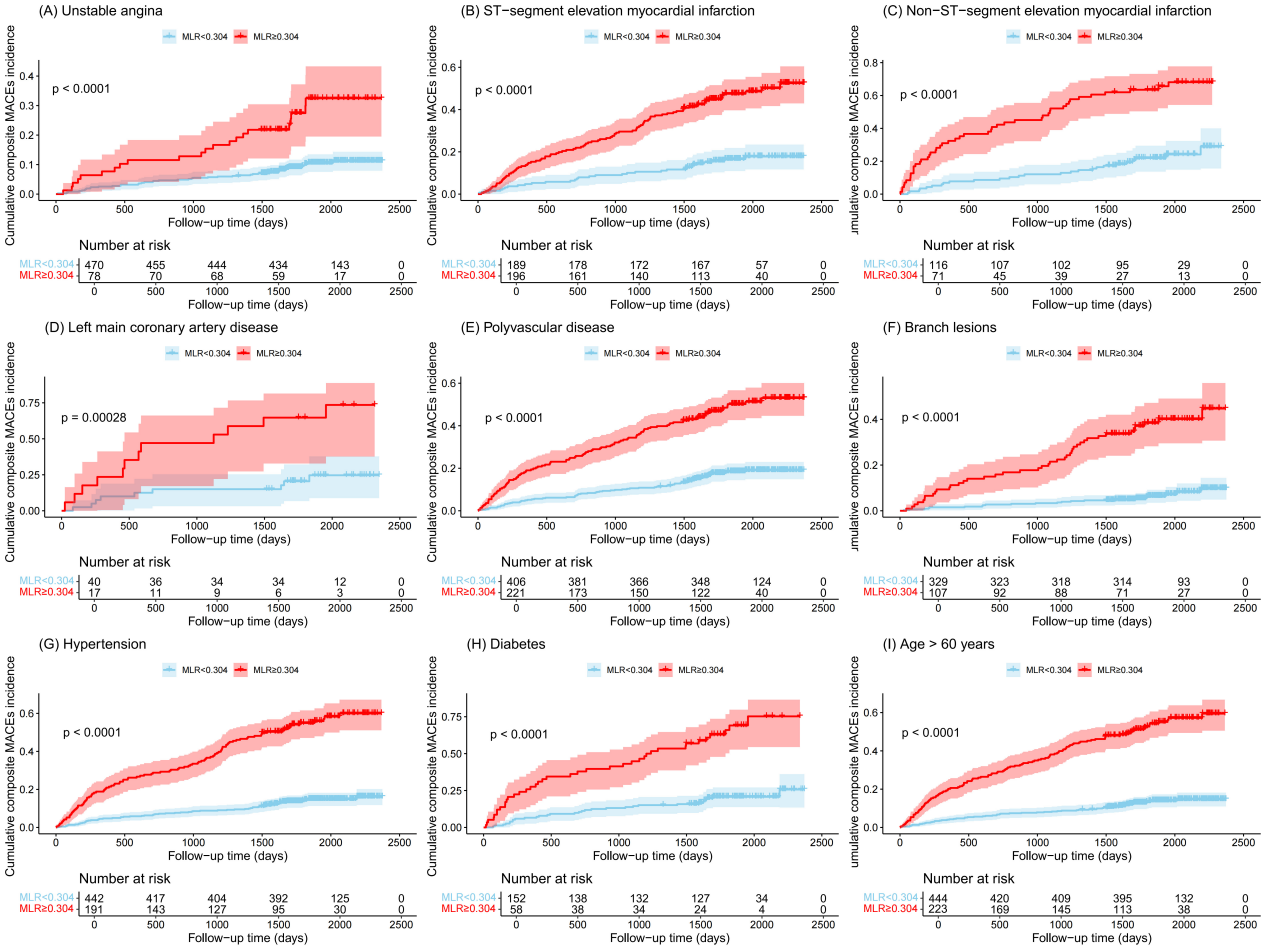
**Cumulative incidences (%) of MACEs stratified by clinical characteristics**. (A) Unstable angina. (B) ST-segment elevation myocardial infarction. (C) Non-ST-segment elevation myocardial infarction. (D) Left main coronary artery disease. (E) Polyvascular disease. (F) Branch lesions. (G) Hypertension. (H) Diabetes. (I) Age >60 years.

Compared with the low-MLR group, the high-MLR group showed a higher cumulative incidence of UA (Fig. 4A), STEMI (Fig. 4B), NSTEMI (Fig. 4C), left main coronary artery disease (Fig. 4D), poly-vascular disease (Fig. 4E), branch lesions (Fig. 4F), hypertension (Fig. 4G), diabetes mellitus (Fig. 4H), and elderly patients (Fig. 4I) (all log-rank *p* < 0.001).

## 4. Discussion

In this study, we assessed the prognostic value of six novel identified lymphocyte-derived inflammatory indices and multiple traditional clinical characteristics in predicting MACEs in newly diagnosed ACS patients. Among these inflammatory indices based on blood cell analysis, the MLR ≥0.304 demonstrated independent associations with MACEs in newly diagnosed ACS patients through multivariate analysis. Notably, the MLR exhibited superior predictive performance compared to traditional clinical characteristics. This indicates its potential clinical value as a prognostic indicator for cardiovascular poor outcomes.

Current pathophysiological theories of coronary artery disease encompass inflammatory cascades, lipid plaque formation, platelet activation, and vascular injury responses [24, 25]. Previous studies have demonstrated that elevated heart rate serves as a critical determinant of adverse clinical outcomes in ACS patients undergoing PCI [26]. Our investigation revealed that both elevated heart rate and systolic blood pressure emerged as significant clinical correlates of MACEs in ACS patients. However, multivariate analysis demonstrated that neither parameter maintained independent predictive value for MACEs occurrence following comprehensive adjustment for established cardiovascular risk factors. The underlying reason might be related to the fact that sustained tachycardia and hypertension can cause damage to arterial endothelial cells. Specifically, at curved or bifurcated arteries, blood flow patterns characterized by oscillatory shear stress promote endothelial transformation into a pro-inflammatory phenotype [27]. This transformation increases cellular inflammation, oxidative stress response, metabolic abnormalities, and endothelial permeability, thereby promoting the occurrence and progression of atherosclerosis [28]. Histopathological analysis of atherosclerotic coronary arteries has revealed that unstable plaques are histologically characterized by infiltration of macrophages, lymphocytes, and mast cells [29]. Notably, activated platelets not only recruit leukocytes but also regulate monocyte migration and subsequent differentiation into macrophages. Atherosclerosis, recognized as a chronic inflammatory disease, progresses through a pathological continuum spanning from endothelial injury, inflammatory cell recruitment, and lipid deposition to eventual plaque rupture. Throughout this disease progression, multiple leukocyte subtypes-including monocytes, neutrophils, and lymphocytes-are actively involved in mediating these pathophysiological transitions [30, 31]. This mechanistic pathway may explain the observed associations between the inflammatory index based on blood cell analysis and adverse outcomes in our cohort study.

Like previous reports [3, 32], older age, hypertension, and diabetes are also risk factors for poor prognosis of ACS. Our study revealed that among ACS patients with advanced age or comorbid diabetes/hypertension, the high-MLR cohort demonstrated significantly elevated MACE incidence compared to their counterparts without these comorbidities. Particularly in three clinically relevant subgroups—geriatric patients (age >60 years), diabetic individuals, and hypertensive cases—MLR measurement exhibited robust capacity for risk stratification, enabling effective prognostic differentiation within these vulnerable populations. MLR, composed of monocytes and lymphocytes, has its earliest traceable record indicating its potential application in the risk stratification of tuberculosis [33]. Subsequently, MLR was studied for conditions such as bipolar disorder [34], chronic kidney disease [35], diabetes [36], and malignant tumors [37, 38, 39], where it can be used to predict poor outcomes. In recent years, the MLR has emerged as a valuable biomarker, finding applications in both the diagnosis and prognosis of cardiovascular diseases [7, 40]. A recent retrospective study showed a significant association between a higher MLR and an increased risk of cardiovascular and all-cause mortality [41]. The meta-analysis showed that MLR was a simple and widely available tool to predict MACEs in patients with CHD [42]. We observed a similar significant association between elevated MLR and mace in patients with newly diagnosed ACS. The difference is that the mean cut-off value in the 19 studies included in the meta-analysis was 0.34, while the cut-off value of MLR in our study was 0.304. In a study that used MLR to predict the mortality of ACS patients, the cut-off value could reach 0.414 [43], which was higher than the cutoff value of MLR in our study. This might be related to the differences in the definitions of MACEs among different studies, as the MACEs in our study included not only death but also four other conditions, such as UA, acute myocardial infarction, etc. Additionally, the smaller sample size might also be the reason for the incomplete consistency of data among different studies. Therefore, currently, the level of evidence regarding the prognostic prediction of the MLR in CHD is generally low, and the optimal threshold remains to be determined.

The immunopathological cascade critically orchestrates atherosclerotic lesion formation and progression, wherein monocytes and lymphocytes emerge as principal mediators of inflammatory pathogenesis. The MLR, calculated from monocytes and lymphocytes, is effective in identifying the presence of vulnerable plaques in ACS patients [44]. Song *et al*. [15] included MLR in the inflammatory prognostic score and reported that a higher score was closely associated with poorer long-term prognosis in patients with ACS undergoing PCI. However, Shumilah *et al*. [45] reported that NLR was the strongest predictor of ACS (*p* < 0.001) and MLR was not a significant predictor of ACS (*p* > 0.05). Gao *et al*. [20] analyzed six novel lymphocyte-based inflammatory indices in predicting MACEs in patients with newly diagnosed CAD, and discovered that only SIIRI showed significantly high predictive performance. Bani *et al*. [21] identified SIRI as a predictor of severe CAD. However, due to potential collinearity among the six lymphocyte-derived inflammatory indices in our study, only MLR was selected. These differences may be because Gao *et al*. [20] defined three events as MACEs, whereas we defined five events as MACEs in this study. Based on the definition of MACEs [22], this study was relatively complete. Our findings suggested that the superior predictive performance of MLR compared to the other five inflammatory indices was context-specific and depended on both the study population characteristics and the clinical definitions of MACEs used in the study. This highlights the need for further validation studies to comprehensively evaluate the prognostic value of MLR across diverse patient populations and varying clinical endpoint criteria. Future studies should focus on elucidating the biological mechanisms underlying the predictive utility of MLR while systematically examining the potential confounding factors that might influence its clinical applicability.

Tanimura *et al*. [46] demonstrated that a history of cancer in ACS patients was independently associated with worse clinical outcomes, including MACEs, compared to those without a cancer history (odds ratio: 4.00, *p* < 0.001). In our study, there was no statistically significant association between cancer history and MACEs in ACS patients. This discrepancy may be attributed to a relatively short follow-up duration in our study. Previous studies have reported that radiation therapy-induced coronary artery disease may manifest 5 to 20 years post-exposure [47]. In the future, we will conduct long-term follow-up to determine whether there is a correlation between the cancer history and MACEs in patients with ACS. Both HDL and LDL are associated with prognosis in the ACS patients [48, 49], which aligns with our observational findings.

Our findings are also in agreement with the results of previously published meta-analyses, which reported significant associations between AF and adverse outcomes in patients with ACS [50, 51]. Current evidence indicates that AF pathogenesis originates from inflammation-mediated myocardial necrosis and fibrotic remodeling [52]. Mechanistically, these structural alterations induce electrophysiological instability through inflammation-induced membrane potential destabilization, which directly facilitates ectopic impulse generation that disrupts the normal rhythm of the heart [53, 54]. Because of the intrinsic pathophysiological interplay between AF and systemic inflammation, AF did not show independent prognostic significance in the multivariable analysis (HR 0.645, 95% CI 0.277–1.503, *p* = 0.309). This suggested that the predictive value of AF may be mediated through inflammatory pathways rather than AF functioning as an autonomous risk determinant. However, Saleh *et al*. [51] suggested that AF served as an independent prognostic indicator for predicting adverse outcomes in ACS patients. This discrepancy may be caused by our study only including newly diagnosed ACS patients, of which only 1.61% were diagnosed with AF. In contrast, 25% of ACS patients included in the study by Saleh *et al*. [51] were previously diagnosed with AF. Therefore, this difference in the inclusion criteria is likely the main contributing factor for the differences in the outcome prediction between the two studies.

This study has several limitations. First, as an observational study, the results merely indicate a correlation rather than a causal relationship. Second, being a single-center retrospective analysis with a relatively small sample size, it may have introduced selection bias and restricted the generalizability of the results. Third, we could not evaluate body mass index as a potential prognostic factor because data for height and weight were not available for a significant proportion of participants. Finally, seasonal variations in blood cell counts could affect the broader applicability of our conclusions. Therefore, larger-cohort multicenter prospective studies are necessary in the future to externally validate our findings and minimize bias through comprehensive clinical data collection.

## 5. Conclusions

Elevated MLR was independently associated with MACEs in patients with newly diagnosed ACS. MLR demonstrated superior predictive performance compared to the other five inflammatory indicators. These findings suggested that MLR is a promising low-cost clinical tool for non-invasive inflammatory monitoring, precise risk stratification, and personalized therapeutic strategies in the management of ACS. The optimal cutoff value of MLR requires further validation through large-scale cohort multicenter studies to establish standardized criteria for the clinical application of these biomarkers.

## Data Availability

The data that support the findings of this study are available on request from the corresponding author.

## Abbreviations

CHD, coronary heart disease; CAD, coronary artery disease; ACS, acute coronary syndrome; UA, unstable angina; STEMI, ST-segment elevation myocardial infarction; NSTEMI, non-ST-segment elevation myocardial infarction; PCI, percutaneous coronary intervention; MACEs, major adverse cardiovascular events; CRP, C-reactive protein; NLR, neutrophil-to-lymphocyte ratio; PLR, platelet-to-lymphocyte ratio; MLR, monocyte-to-lymphocyte ratio; SII, systemic immune-inflammation index; SIRI, systemic inflammation response index; SIIRI, systemic immune-inflammatory response index; HR, hazard ratio; CI, confidence interval; HF, heart failure; AF, atrial fibrillation; LDL, low-density lipoprotein; HDL, high-density lipoprotein; ROC, receiver operating characteristic; AUC, area under the curve.

## Supplementary Material

Supplementary material associated with this article can be found, in the online version, at https://doi.org/10.31083/RCM39917.
