# Supplementary material for: The Monocyte-to-Lymphocyte Ratio Exhibits A Superior Prognostic Value in Patients with Newly Diagnosed Acute Coronary Syndrome
Source: Rev Cardiovasc Med. 2025 Oct 30;26(10):39917. doi: 10.31083/RCM39917 (PMC12593858; doi:10.31083/RCM39917)
Supplement: Supplementary file 1 [file 2153-8174-26-10-39917-s1.doc]

Supplementary material


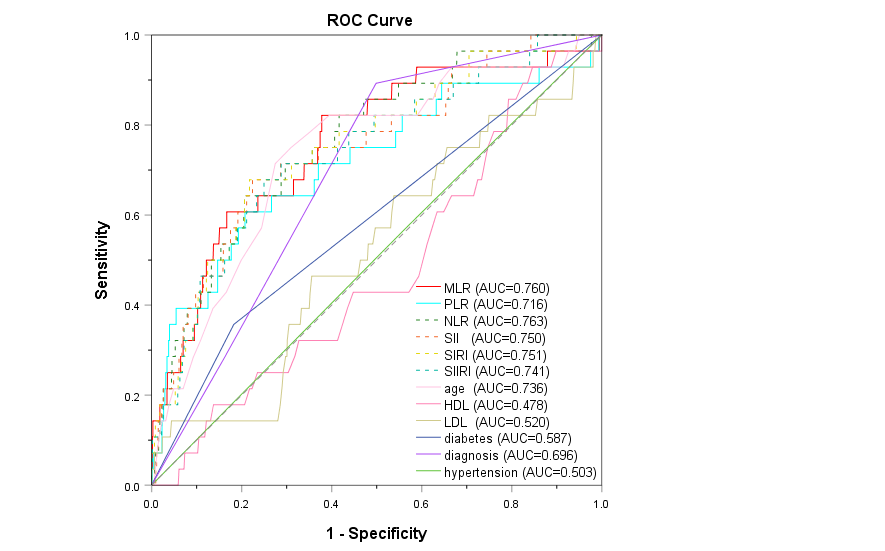


Supplementary Fig.1. ROC curves of inflammatory biomarkers for predicting heart failure.

Supplementary Table 1. Diagnostic performances of various biomarkers in predicting heart failure among ACS patients.

| Model | Cut-off value | *p*-value | AUC (95% CI) | SEN | SPE | PPV | NPV |
| --- | --- | --- | --- | --- | --- | --- | --- |
| Age | 68 | ＜0.001 | 0.736 (0.710-0.762) | 0.750 | 0.691 | 0.063 | 0.990 |
| Age | 65 | ＜0.001 | 0.713 (0.686-0.740) | 0.821 | 0.605 | 0.051 | 0.992 |
| Hypertension | Yes | 0.947 | 0.503 (0.474-0.533) | 0.571 | 0.435 | 0.025 | 0.975 |
| Diagnosis | MI | ＜0.001 | 0.696 (0.666-0.721) | 0.893 | 0.499 | 0.044 | 0.995 |
| Diabetes mellitus | Yes | 0.612 | 0.587 (0.558-0.616) | 0.357 | 0.816 | 0.048 | 0.980 |
| MLR | 0.267 | ＜0.001 | 0.760 (0.733-0.784) | 0.821 | 0.621 | 0.052 | 0.987 |
| HDL | 1.15 mmol/L | 0.677 | 0.478 (0.456-0.552) | 0.571 | 0.571 | 0.029 | 0.980 |
| LDL | 2.73 mmol/L | 0.726 | 0.520 (0.490-0.550) | 0.143 | 0.721 | 0.027 | 0.976 |

Abbreviations: AUC, area under the curve; CI, confidence interval; SEN, Sensitivity; SPE, Specificity; PPV, positive predictive value; NPV, negative predictive value; MI, myocardial infarction; MLR, monocyte-to-lymphocyte ratio; HDL, High-density lipoprotein; LDL, Low-density lipoprotein.
